# Prey and Venom Efficacy of Male and Female Wandering Spider, *Phoneutria boliviensis* (Araneae: Ctenidae)

**DOI:** 10.3390/toxins11110622

**Published:** 2019-10-27

**Authors:** Juan Carlos Valenzuela-Rojas, Julio César González-Gómez, Arie van der Meijden, Juan Nicolás Cortés, Giovany Guevara, Lida Marcela Franco, Stano Pekár, Luis Fernando García

**Affiliations:** 1 Grupo de Investigación Biología y Ecología de Artrópodos (BEA), Corporación Huiltur y Facultad de Ciencias, Universidad del Tolima, Altos de Santa Helena, Ibagué 730001, Colombia; juanbioquimico@gmail.com (J.C.V.-R.); gonzalezgomez40@gmail.com (J.C.G.-G.); mail@arievandermeijden.nl (A.v.d.M.); 2 CIBIO Research Centre in Biodiversity and Genetic Resources, InBIO, Universidade do Porto, Campus Agrário de Vairão, Rua Padre Armando Quintas 7, 4485-661 Vairão, Vila do Conde, Portugal; 3 Facultad de Ciencias Naturales y Matemáticas, Universidad de Ibagué, Carrera 22 calle 67, Ibagué 730001, Colombia; c120132010@estudiantesunibague.edu.co (J.N.C.); lida.franco@unibague.edu.co (L.M.F.); 4 Grupo de Investigación en Zoología, Facultad de Ciencias, Universidad del Tolima, Altos de Santa Helena, Ibagué 730001, Colombia; ggcolombia@gmail.com; 5 Department of Botany and Zoology, Faculty of Science, Masaryk University, Kotlářská 2, 61137 Brno, Czech Republic; pekar@sci.muni.cz; 6 Grupo Multidisciplinario en Ecología para la Agricultura, Centro Universitario Regional del Este, Treinta y Tres 33000, Uruguay

**Keywords:** venom, toxins, LD50, trophic niche, sexual dimorphism

## Abstract

Spiders rely on venom to catch prey and few species are even capable of capturing vertebrates. The majority of spiders are generalist predators, possessing complex venom, in which different toxins seem to target different types of prey. In this study, we focused on the trophic ecology and venom toxicity of *Phoneutria boliviensis* F. O. Pickard-Cambridge, 1897, a Central American spider of medical importance. We tested the hypothesis that its venom is adapted to catch vertebrate prey by studying its trophic ecology and venom toxicity against selected vertebrate and invertebrate prey. We compared both trophic ecology (based on acceptance experiments) and toxicity (based on bioassays) among sexes of this species. We found that *P*. *boliviensis* accepted geckos, spiders, and cockroaches as prey, but rejected frogs. There was no difference in acceptance between males and females. The venom of *P*. *boliviensis* was far more efficient against vertebrate (geckos) than invertebrate (spiders) prey in both immobilization time and LD50. Surprisingly, venom of males was more efficient than that of females. Our results suggest that *P. boliviensis* has adapted its venom to catch vertebrates, which may explain its toxicity to humans.

## 1. Introduction

Venoms are substances injected into another organism with the aim of altering its state to the benefit of the injecting organism. Chemically, venoms are mixtures of different peptide and protein toxins [1]. Animal venoms are mainly used for prey capture, feeding, and defensive purposes [2]. For venomous predators, the use of venom is a highly efficient strategy as it allows them to paralyze prey, reducing its possible escape or retaliation [3], or to partially digest it, facilitating the feeding processes [4]. For example, *Conus* snails have a very potent venom for capturing fast and mobile prey such as fish [5], while some snakes have developed highly specific toxins to subdue different prey types such as arthropods, birds, or reptiles [6,7,8,9,10].

Spiders are known as the most diverse group of terrestrial predators [11], and although some groups lack venomous glands [1,12], most species rely on venom to catch prey [13]. Pekár et al. [14] showed that strict specialist spiders, like the araneophagous and myrmecophagous species, have venom components, which might act specifically against their preferred prey when compared to generalist species. In contrast, generalist species have a rich chemical cocktail venom with toxins that affect different prey types [1]. For example, in the medically important *Latrodectus* spiders, toxins target different prey such as crustaceans, insects, and vertebrates [1]. Nevertheless, studies about venom composition and its role in prey capture in generalist spiders are still scarce.

Vertebrate predation by spiders remains an uncommon phenomenon overall. However, it appears to be relatively frequent in some spider families, which include large-sized groups such as Theraphosidae, Ctenidae, Lycosidae, and Pisauridae, among others [15]. Vertebrates consumed by spiders include small mammals like bats, mice, or small marsupials [15,16,17,18]. Reptiles, including lizards and snakes, are also consumed [15,19]. Anurans are the most frequent prey among amphibians [18,20], but some species also consume fish and birds [15,21]. Although it has been suggested that toxins in generalist spiders are complex enough to similarly affect both vertebrates and invertebrates [1], specific venom components for targeting vertebrates as prey have been reported in some groups such as Mygalomorphs and black-widow and some Therididae (e.g., widows of the genera *Latrodectus* and *Steatoda*) [1,22,23].

Spiders of the genus *Phoneutria* represent one of the main groups of medically important spiders in South America because of their defensive behavior, anthropogenic habits, and potent venom [16,24,25]. Toxicity of these spiders also varies with sex, as it does in other spider species [26], where females are more toxic than males [27]. Studies on the toxicity of *Phoneutria* spiders have shown that some of their venom components are highly toxic against insects and vertebrates [28]. Although toxicity to insects has been suggested to be a mechanism for prey capture [29], ecological and evolutionary causes for high toxicity in vertebrates have been poorly explored. Recent records in the diet of *Phoneutria boliviensis*, suggest these spiders prey on several arthropod species but also consume vertebrates, mainly reptiles and anurans [30]. In addition, mammals and birds have occasionally been reported as prey in other *Phoneutria* spiders [16]. As a consequence, we hypothesize that just like in *Latrodectus* spiders, high toxicity to vertebrates in *Phoneutria* spiders might be a consequence of feeding on this kind of prey. However, to our knowledge, there are no studies that have analyzed the trophic ecology of *Phoneutria* spiders linking it with its toxicity against different prey types.

Knowledge on trophic ecology is essential for understanding the functional role of the venom since a more realistic effect can be measured when natural prey is used. Therefore, the aim of this study was to test the hypothesis that *P. boliviensis*, a wandering and medically important spider with anthropic habits, captures both arthropods and vertebrate prey and has adapted its venom for such prey. Since, in spiders, females tend to be larger and feed more frequently than males [31], we evaluated whether prey acceptance and venom efficiency varied intersexually. We also hypothesized a higher acceptance, and in consequence, more toxicity in females against consumed prey.

## 2. Results

### 2.1. Prey Acceptance and Immobilization Time

**Figure 1:**
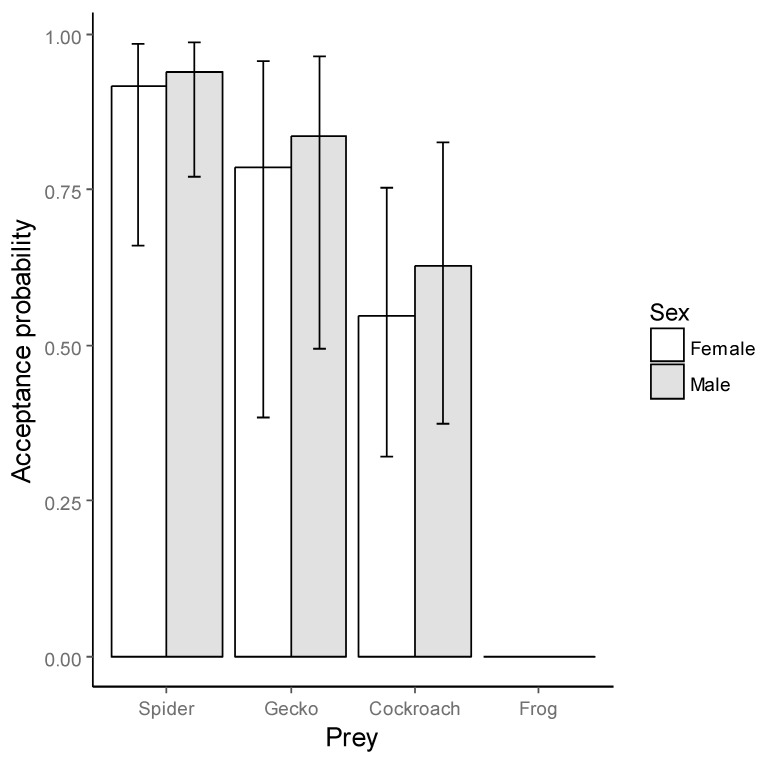
Comparison of the probability of acceptance of four prey types (*Periplaneta Americana* cockroaches, *Hemidactylus frenatus* geckos, and *Spinoctenus* sp. spiders) by females (n = 20) and males (n = 20) of *Phoneutria boliviensis.* Bars are means; whiskers are confidence intervals.

We found that three out of four prey types were accepted (i.e., killed and consumed), namely spiders *(Spinoctenus* sp.), geckos (*Hemidactylus frenatus*), and cockroaches (*Periplaneta americana*) by males and females of *P. boliviensis* (Figure 1). The frog *Engystomops pustulosus* Lynch, 1970 was rejected in all trials (Figure 1, Table S1); although in five instances (12.5%), spiders attacked the frog. Bitten frogs always died, yet they were never consumed, in contrast to the remaining prey types. We did not find significant differences in the acceptance of prey between sexes (generalized estimating equation (GEE)-b, *X*^2^_1_ = 0.50, *p* = 0.45) or significant effect of weight on acceptance (GEE-b, *X*^2^_1_ = 0.50, *p* = 0.49), but a difference was observed among prey types (GEE-b, *X*^2^_3_ = 5302.20, *p* < 0.0001), with a lower acceptance of the frog compared to the other prey types.

**Figure 2:**
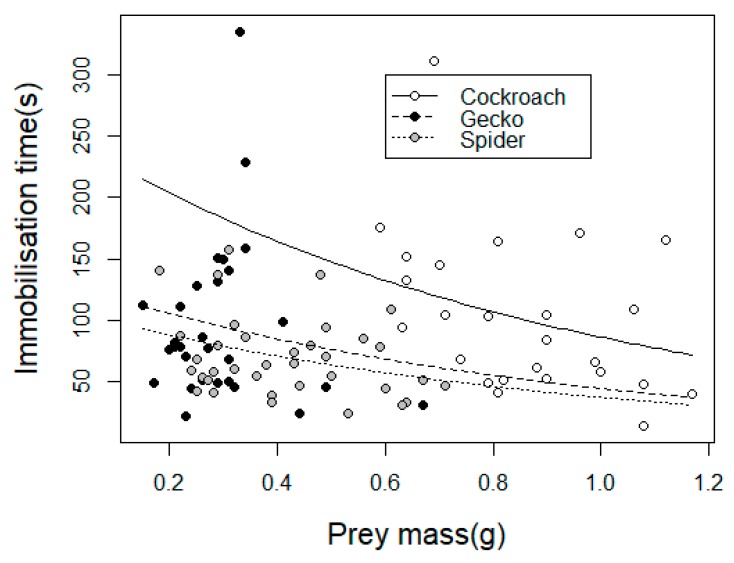
Relationship between mass of prey and immobilization time of three prey types, namely cockroaches (*Periplaneta americana),* geckos (*Hemidactylus frenatus*), and spiders (*Spinoctenus* sp.) by *P. boliviensis*. Estimated exponential models are shown.

The immobilization time was not significantly different between the sexes (GEE-g, *X*^2^_1_ = 0.78, *p* = 0.37). However, we found that overall immobilization time was significantly different among prey types (GEE-g, *X*^2^_2_ = 10.16, *p* = 0.003): Cockroaches were immobilized in significantly longer times than the other prey (contrasts, *p* < 0.01, Figure 2). We found a significant effect of prey mass on immobilization times (GEE-g, *X*^2^_1_ = 8.62, *p* = 0.0004). 

Number of bites was not significantly different among prey types (GEE-p, *X*^2^_1_ = 1.03, *p* = 0.60) or sex (GEE-p, *X*^2^_1_ = 1.19, *p* = 0.28) and was not affected by mass (GEE-p, *X*^2^_1_ = 0.24, *p* = 0.62). Overall *P. boliviensis* made 1.42 (SE = 0.09) bites per prey (Table S1).

### 2.2. Venom Volume

**Figure 3:**
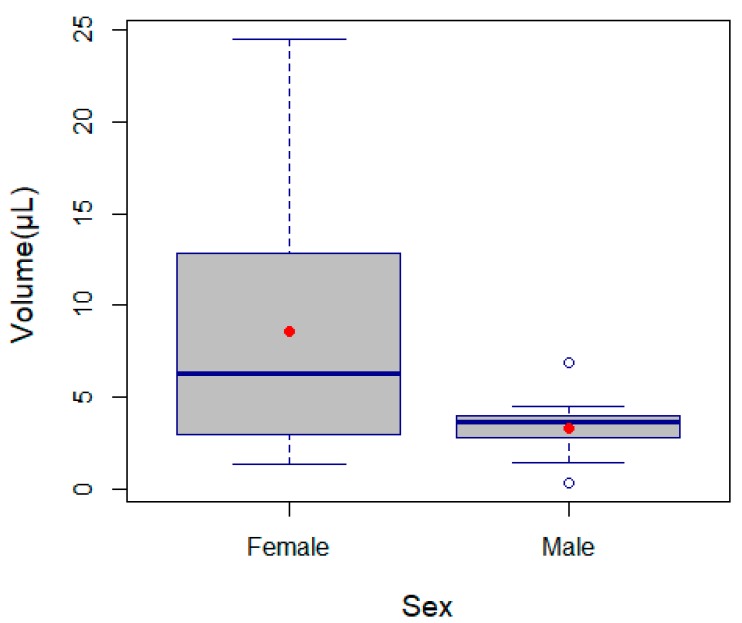
Boxplot showing comparison of venom volume produced by females and males of *Phoneutria boliviensis*. Thick lines are medians, boxes are quartiles, and whiskers are 1.5 times interquartile range. Red points represent means.

We did not find a significant effect of spider size (prosoma length) on venom production (LM, F_1, 29_ = 3.70, *p* = 0.06), however we found significant differences between sexes (LM, F_1, 29_ = 7.36, *p* = 0.0012): Females produced more venom (mean ± standard error: 8.60 ± 1.53 µL) than males (3.31 ± 0.38 µL) (Figure 3 and Table S2).

### 2.3. Toxicity

Symptoms observed after injecting different venom concentrations (see Table S3) included erratic movements, paralysis, and leg curl in spiders, while in geckos, we observed them running in circles in the container, repeatedly opening the mouth, and displaying leg paralysis before the total paralysis. No mortality was recorded in the control groups for spiders and geckos.

**Figure 4:**
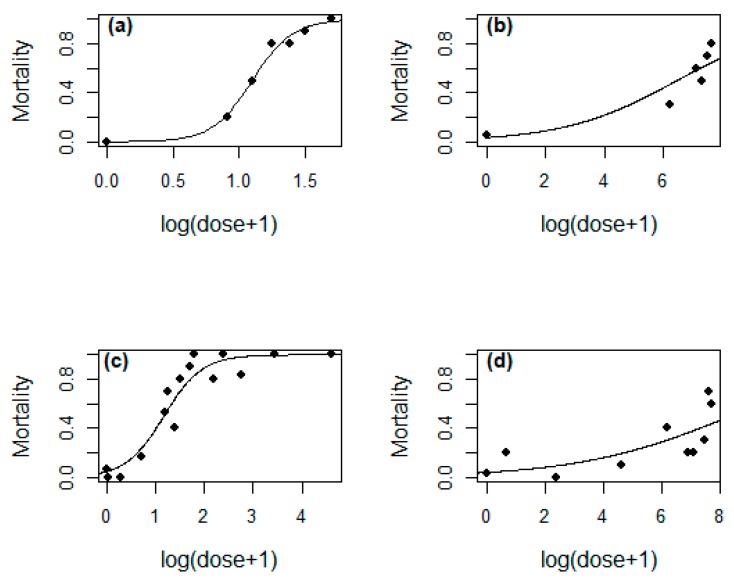
Relationships between dose and mortality after venom injection of *Phoneutria boliviensis* for males against (**a**) gecko (*Hemidactylus frenatus)* and (**b**) spider (*Spinoctenus* sp.) prey and females against (**c**) gecko (*Hemidactylus frenatus*) and (**d**) spider prey (*Spinoctenus* sp.). Estimated logit models are shown.

We found a significant interaction between the prey types and dose (GLM-b, *X*^2^_1_ = 73.16, *p* < 0.0001, Figure 4). Similarly, mortality was significantly different between prey (GLM-b, *X*^2^_1_ = 18.35, *p* < 0.0001), but not between sexes (GLM-b, *X*^2^_1_ = 3.34, *p* = 0.06). 

Consequently, the LD_50_ values for each prey and sex were different (Table 1).

## 3. Discussion

Our results show that *P. boliviensis* captures both invertebrate and vertebrate prey. Males and females of *P. boliviensis* display a similar prey acceptance, as both captured and consumed the same prey. *Phoneutria boliviensis* accepted and was able to overcome animals with different morphologies, such as spiders, cockroaches, and geckos, suggesting this species is likely an euryphagous species [32]. These results thus confirm ability to exploit small reptiles as has been previously reported for this genus from the field [30]. Rejection of frogs was an unexpected result since anurans are readily consumed by *Phoneutria* spiders [20,33]. However, it has been reported that the frog *E. pustulosus* possesses defensive glands, which store noxious substances [34], which may have contributed to their avoidance by the spiders. This is supported by observations as the rejection occurred only after the spider bit the frog.

When evaluating the immobilization times, we found them to be similar for both male and female spiders. However, immobilization time was different between the offered prey types: Spiders were the most susceptible, while cockroaches were the least. Shorter times observed for spiders might be achieved by injection of more venom. Araneophagy is a risky behavior [35] and is used when predators possess effective weapons. According to the venom optimization hypothesis, venomous animals are able to regulate volume of administered venom according to some prey traits, and this might be the case when attacking other spiders [36]. Short immobilization times in geckos might be explained by the fact that it is a fast prey that needs to be paralyzed quickly to prevent escape. 

In particular, the cockroaches exhibited the longest immobilization times, probably due to their cuticular armor, which in some cases prevented the spider from biting some regions, such as the abdomen and the ventral part of thorax. Alternatively, cockroaches might be more resistant to *P. boliviensis* venom. Overall, we found that *P. boliviensis* needed a single bite to immobilize prey, probably as the spider was able to grasp and inject venom at the same time. A similar trend has been observed in *Loxosceles*, another medically important species [37]. Surprisingly, we observed an inverse relationship between prey mass and immobilization time, which contrasts with previous records [38]. We hypothesize that this could be due to the fact that larger prey were more active than smaller prey, which might cause the spider injects more venom; a similar trend has been shown in scorpions where sting use is directly related to prey activity [39] or centipedes where prey size selection is related to venom availability [40].

The volume of the venom was higher in females than in males, which is not surprising, since in general in spiders, females produce more venom than males [41,42,43]. A similar difference, observed in *Phoneutria nigriventer* Keyserling, 1891, was attributed to allometry [27], which is not supported by our study. We also observed a higher variation of volume venom in females when compared to males. We assume this variation might be a consequence of venom production, which although on average was higher in females, caused a higher variation when some individuals produced a low quantity. This variation would not be so evident in males, where the venom production was much lower compared to females. However, not only venom volume varied between sexes in *P. boliviensis*, but also toxicity varied, which might be either due to differences in venom compounds or in the concentrations of the same compounds [44]. We expected a higher toxicity in females, in agreement with former studies in other *Phoneutria* spiders [27]. Instead, males had slightly more toxic venom to geckos and much more toxic venom to spiders when compared to females. However, in some mygalomorph species, such as *Hadronyche (Atrax)*, males have also been reported to be more toxic than females [25]. We attribute these differences to the use of different prey, as it has been shown that venom can act in a different way in closely related organisms and might be biased when not using real prey [9]. This is supported by a significant interaction between sex and prey type in the toxicity. Apparently, there is a trade-off between the venom volume and its efficacy: Males achieved high toxicity at lower dose while females at higher doses. A selection in toxicity for different defensive purposes is unlikely in this species as both males and females are equally exposed to potential vertebrate predators given their wandering habits. In addition, both sexes readily consumed reptiles such as geckos, which emphasize the important role of venom when capturing this kind of prey.

Venom is strongly linked to prey capture in spiders and other arthropods. For example, it has been suggested that prey-specialized spiders have specific toxins effective for subduing their preferred prey [14]. In the case of larger spider species, vertebrate specific toxins might have an important role in prey capture. For example, theraphosid spiders possess toxins highly effective against small terrestrial vertebrates, which might be attributed for prey capture [45], a similar case occurs in belostomatid bugs, whose toxins can subdue small aquatic vertebrates such as fishes, amphibians, and reptiles [46]. The higher toxicity of *P. boliviensis* to geckos suggests it plays an important role in the capture of this kind of prey. We cannot dismiss the possibility that the venom of *P. boliviensis* is also used for defensive purposes against vertebrates. Interestingly, although vertebrate-specific toxins are present in *P. nigriventer* individuals from early stages [47,48], lethality to vertebrates, namely mice, occurs mainly when spiders reach adulthood [41]. This might be explained by adults administering higher venom volume than juveniles or it might be due an ontogenic shift in toxin production, behavior, and overall venom toxicity.

The LD_50_ values of *P. boliviensis* against geckos are slightly higher than those reported for other *Phoneutria* species (Table 2) or against mice (Table 2), suggesting that *Phoneutria* venom might act similarly in several vertebrate species including mammals. A similar trend is observed in several *Latrodecus* spiders [49], whose LD_50_ values against mouse are slightly smaller than those reported here for *P. boliviensis*, an expected result given the vertebrate feeding habits recorded in *Latrodectus* spiders [50]. However, comparison should be carefully interpreted as mice have not been used here and these are not natural prey for *P. boliviensis*. [30].

The evidence gathered in this study suggests that high toxicity of *Phoneutria* venom against vertebrate prey might be a consequence of feeding on this prey type. Therefore, more studies on the feeding ecology of *Phoneutria* spiders are required to understand the evolution of venom composition as well as venom use and optimization against different prey types. Future studies should also focus on the feeding and defensive behavior of other *Phoneutria* species and other related vertebrate-eating spiders, such as *Ctenus* and *Ancylometes* [20].

## 4. Materials and Methods 

### 4.1. Specimen Collection and Housing

We collected 50 adult individuals (25 males, 25 females) of *P. boliviensis* in the locality of Oporapa, Colombia (2° 1’ 40.5" N; 75° 59’ 43" W). Specimens were sampled by hand at night using a headlamp, in grassland and coffee plantations. Collected specimens were placed singly in 710 mL plastic containers and transferred to the biology laboratory at the Universidad de Ibagué, where they were kept individually in plastic terraria (23 × 17 × 14 cm). A piece of curved cardboard was placed inside the terrarium as a shelter, a piece of wet cotton was provided for humidity. Water was provided ad libitum, moistening a cotton every two days with 5 mL of water. Conditions at the sampling locality, i.e., humidity (80 ± 10%), temperature (25 ± 1 °C), and photoperiod (12:12 h, light:dark), were simulated with a thermoregulator and a humidifier installed in the laboratory. Voucher specimens are deposited in the zoology collection at the Universidad del Tolima, Ibagué. 

Four prey species were used, two vertebrates and two invertebrates. Prey species were selected based on 1) their sympatry and abundance at the same site as *P. boliviensis* and 2) evidence of the consumption of closely related prey species by other *Phoneutria* or related ctenid spiders. As vertebrate prey, we selected the gecko *Hemidactylus frenatus* Duméril & Bibron, 1836 (mass (mean ± SE): 0.29 ± 0.01 g, size (mean ± SE): 47.57 ± 1.25)) and the frog *E. pustulosus* (mass: 1.26 ± 0.37 g, size: 24.27 ± 0.54), which were reported to be preyed upon by *Phoneutria* spiders [30] and other ctenid spiders [52], respectively. As arthropod prey, we used the spider *Spinoctenus* sp. (Araneae, Ctenidae, mass: 0.41 ± 0.02 g, size: 15.95 ± 0.61) and the cockroach *Periplaneta americana* Linnaeus, 1758 (mass: 0.85 ± 0.02 g, size: 29.17 ± 0.95), based on reports of Lucas [53] and Hazzi [54]. All prey were collected next to buildings and gardens of the Universidad de Ibagué and the Universidad del Tolima. All prey specimens were maintained ad libitum by supplying *Tenebrio molitor* Linnaeus, 1758 every two days (*Periplaneta americana* with a diet of oats and carrots). *Spinoctenus* sp. were kept singly in 148 mL containers, while the other prey were kept together in 50 × 50 × 50 cm containers, under the same environmental conditions as *P. boliviensis*. Vertebrates used in this study (geckos and frogs) were kept under laboratory conditions according to international standards [55].

### 4.2. Prey Acceptance and Immobilization

Before using spiders in the acceptance experiment, we standardized their hunger level by feeding all spiders with *T. molitor* [56] larvae until the spider stopped feeding, so it was considered satiated. Subsequently all individuals were deprived of prey for 12 days before starting the experiment in order to increase their capture success rate. We randomly assigned one prey species to a spider (using the Excel pseudorandom function). We repeated this procedure until all selected prey had been offered to all spiders, namely 20 males: (mean ±SE, weight: 2.09 ± 0.38 g, prosoma length: 12.85 ± 0.48 mm) and 20 females (weight: 2.41 ± 0.15 g, prosoma length: 13.59 ± 0.47 mm), in order to achieve a complete block design [57].

Spiders were individually placed in an observation arena of 23 × 17 × 14 cm 2 h before starting the experiment. Trials were performed at night, as the spiders are nocturnal. For this purpose, recordings were made with a NIKON© D3300 camera under red light, since this color is not perceived by spiders [58]. All prey and spiders were weighed on a Precisa© analytic balance model LX 220A, with 0.01 mg precision. A prey was released to the arena housing a spider and its fate was observed for 10 min. If the prey was attacked and consumption of the prey had started during that time, it was classified as accepted. If the prey was attacked but not killed or killed but not consumed it was classified as rejected. For all successful captures, we recorded the immobilization time, defined as the time (in seconds) between the first bite and when the prey stopped moving.

Since the experiment had a block design (due to repeated measurements), data were analyzed by generalized estimating equations (GEE) from the geepack [59], which is an extension of generalized linear models (GLM) for correlated data [60]. In the case of acceptance, we used a binomial distribution (GEE-b), while in the case of immobilization time, we used a Gamma distribution (GEE-g). In the linear predictor, spider specimen was considered as a block and prey type and prey mass were used as explanatory variables. We compared the number of bites between prey and sex using GEE with Poisson distribution, using the same explanatory variables as in immobilization analysis, and number of bites as the response variable.

### 4.3. Venom Extraction and Volume

The venom of 15 other males (mean ± SE, prosoma length: 12.43 ± 0.39 mm) and 18 females (mean ± SE, prosoma length: 13.42 ± 0.38 mm) of *P. boliviensis* was extracted. For this, we built a device to immobilize spiders without anesthetizing them [61], since it has been shown that anesthesia can increase mortality or alter the behavior and physiology of the animal [62]. The designed device was a plastic container covered with a fine plastic mesh and the spider was pressed against the mesh with the help of a foam piece (Figure S1).

We used an ENTES electro-stimulator (Entomopixel™ Company: www.entomopixel.com) set at a square wave with an amplitude of 12 V, a frequency of 20 Hz, and a 10% duty factor. Spider electro-stimulation was done by placing the electrodes on the prosoma and cheliceral base for 5 s, electrodes were moved at different positions to get as much venom as possible. Electrostimulation method was chosen based on its efficiency for extracting venom in large spiders [63]. At the same time, two glass capillary tubes (internal diameter (mean ± SE): 1.16 ± 0.004 mm) were placed over the tips of the fangs to collect the venom and prevent possible contamination from other fluids the spider may expel. Spiders did not appear to suffer ill effects of electrostimulation, and were maintained healthy in the lab after venom extraction. The capillaries were then photographed with a size reference in order to estimate the volume, and the venom was transferred to a low protein binding cryotube and flash-frozen in liquid nitrogen. The extracted venom was lyophilized, weighed by Precisa © analytical balance, and stored in a freezer at −85 °C [24] until use. The volume of venom was calculated from the images by measuring the length and diameter of the fluid column. The mean of venom volumes obtained from each fang was used in the analysis. Venom volume between males and females was compared using a linear model (LM). The linear predictor included spider size and sex. 

### 4.4. Toxicity Bioassays

We used one vertebrate and one arthropod prey species in the bioassays, namely the gecko *H. frenatus* (2.62 ± 0.34 g) and the spider *Spinoctenus* sp (0.84 ± 0.22 g); these prey were selected as they were accepted at higher frequency by *P. bolivinensis* than other prey. The geckos were kept according to the international standards for the use of reptiles in laboratory investigations [54]. In vivo experiments were approved by the ethical committee of the Universidad de Ibagué (001 10 November 2017). Reports for gecko results on this study followed the Animal Research: Reporting of In Vivo Experiments (ARRIVE) guidelines [64]. Both *Spinoctenus* sp. spiders (n = 190) and geckos (n = 259) used were adults. The *Spinoctenus* sp. spiders were fed ad libitum with *T. molitor* larvae and geckos were fed with juvenile individuals of the cricket *Acheta domestica* Linnaeus, 1758 weekly during two weeks before the bioassays. The temperature, relative humidity, and conditioning photoperiod were similar to that of spiders (temperature = 25 ± 1 °C, relative humidity = 80 ± 10%, and photoperiod = 12 h light:12 h dark). Individuals were weighed before using them in bioassay to the nearest 0.1 mg.

The lyophilized venom was diluted in physiological saline solution [27,48]. Individuals were randomly assigned to each experimental group. A different number of prey animals were used in our bioassay because of the difference in availability of female and male venom (Table S1). The venom solution was injected with a 10 μL Hamilton© syringe. The geckos were held in hand and injected subdermally through the skin, in the left rear leg. We selected this location as no vital organs could be affected, similar to as has been done in mice [27]. Individuals of control group were injected only with physiological saline solution. After the injections, the geckos were placed individually in 710 mL plastic containers. In the case of *Spinoctenus* sp. spiders, these were fixed using the same mechanism as with *P. boliviensis* in milking, and were injected into the joint section between coxae and leg IV, avoiding any possible contact with the sternum so no vital organs would be affected. After the injections, the spiders were placed individually in 148 mL plastic containers. All injections were made by the same person (J.C.V.R) to avoid experimenter bias. Once injected, all animals were kept under same conditions as described on the prey maintenance section. Water was provided but not food.

Treated animals were immediately checked after being injected and rechecked 24 h later. Spiders were considered dead after 24 h if they could not turn around when turned side up. In the case of the geckos, the same procedure was performed while also checking for the absence of respiration.

Data on survival were compared between sex and prey using GLM with a binomial distribution and logit link [65]. The dose was logarithmically transformed [66]. We used sex and prey type as explanatory variables, and mortality as the response variable. All the statistical analyses were carried out with R software version 3.5.0 [67], LD_50_, and its SE was estimated using a function from the MASS package [68].

## Figures and Tables

**Table 1:** Estimated LD_50_ values (mg/kg) for two different prey (gecko, spider) of females and males of *P. boliviensis;* 95% confidence intervals for the means are given in brackets.

PREY	SEX	
	Female	Male
Gecko	2.19 (1.57, 2.96)	2.03 (1.92, 2.16)
Spider	4229 (460, 38,745)	639 (248, 1636)

**Table 2:** Comparison of LD_50_ (mg/kg) of spiders of the genus *Phoneutria* for different prey types. Sex: M—male, F—female, M/F: Pooled male and female venom. * Values reported in this study.

Species	Prey					
	Sex	Mouse	Dog	Spider	Gecko	Fly
*P. nigriventer* [27]	F	0.63	-	-	-	-
*P. nigriventer* [27]	M	1.57	-	-	-	-
*P. nigriventer* [48]	M/F	0.6	-	-	-	22.40
*P. keyserlingi* [48]	M/F	0.9	-	-	-	
*P. reidyi* [48]	M/F	0.11	-	-	-	0.85
*P. fera* [51]	M/F	0.76	0.20	-	-	-
P. boliviensis *	M	-	-	639	2.03	-
P. boliviensis *	F	-	-	4229	2.20	-

## Supplementary Materials

The following are available online at https://www.mdpi.com/2072-6651/11/11/622/s1, Figure S1: Device built for spider milking, with foam holding the immobilized spider. Table S1: Record for behavioral observations, including the explanatory variables (acceptance, immobilization time, number of bites) of males and females of *P. boliviensis* when attacking cockroaches (*Periplaneta americana*), frogs (*Engystomops pustulosus*), geckos (*Hemidactylus frenatus*) and spiders (*Spinoctenus* sp.). Table S2. Prosoma length and mean volume produced by males and females of *P. boliviensis*. Table S3. List of doses used the in the bioassays for females (F) and males (M) of *P. boliviensis* and two prey types, gecko (*Hemidactylus frenatus*) and spider (*Spinoctenus* sp.).
