# Supplementary material for: Prey and Venom Efficacy of Male and Female Wandering Spider, Phoneutria boliviensis (Araneae: Ctenidae)
Source: Toxins (Basel). 2019 Oct 27;11(11):622. doi: 10.3390/toxins11110622 (PMC6891708; doi:10.3390/toxins11110622)
Supplement: Supplementary file 1 [file toxins-11-00622-s001.pdf]

Supplementary Materials: Prey and Venom Efficacy of Male and Female Wandering Spider, Phoneutria Boliviensis (Araneae: Ctenidae).

Juan Carlos Valenzuela-Rojas, Julio César González-Gómez, Arie van der Meijden, Juan Nicolás Cortés, Giovany Guevara, Lida Marcela Franco, Stano Pekár and Luis Fernando García

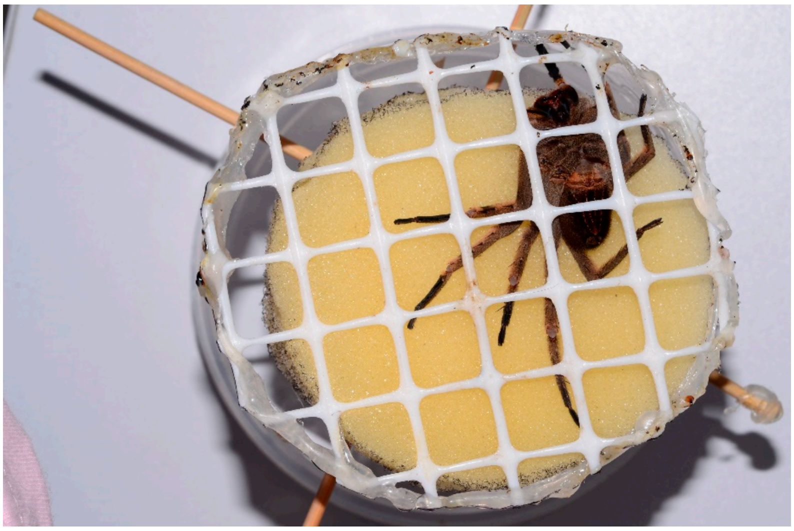

Figure S1. Device built for spider milking, with foam holding the immobilized spider.

Table S1. Record for behavioral observations, including the explanatory variables (acceptance, immobilization time, number of bites) of males and females of *P. boliviensis* when attacking cockroaches (*Periplaneta americana),* frogs (*Engystomops pustulosus*), geckos (*Hemidactylus frenatus*) and spiders (*Spinoctenus* sp.). (na: not available data).

Sex	ID	Prey	Acceptance	Spider Mass(g)	Prey Mass(g)	Spider Prosoma Length(cm)	Immobilization Time(s)	Number of Bites
Male	1Male	Frog	0	1.82	1.02	13.14	na	na
Male	1Male	Gecko	1	1.97	0.30	13.14	150	1
Male	1Male	Spider	1	1.99	0.44	13.14	47	1
Male	1Male	Cockroach	0	1.82	0.73	13.14	na	na
Male	2Male	Cockroach	1	2.47	0.64	14.97	133	1
Male	2Male	Spider	1	2.44	0.49	14.97	71	2
Male	2Male	Gecko	1	2.48	0.31	14.97	68	1
Male	2Male	Frog	0	2.25	1.90	14.97	na	na
Male	3Male	Spider	1	2.67	0.29	15.96	80	1
Male	3Male	Frog	0	2.43	0.95	15.96	na	na
Male	3Male	Cockroach	0	2.43	0.75	15.96	na	na
Male	3Male	Gecko	0	2.43	0.29	15.96	na	na
Male	4Male	Frog	0	2.21	1.33	14.87	na	na
Male	4Male	Gecko	1	2.34	0.33	14.87	335	1
Male	4Male	Spider	1	2.39	0.31	14.87	158	2
Male	4Male	Cockroach	0	2.09	0.74	14.87	na	na
Male	5Male	Cockroach	0	2.48	0.74	14.42	na	na
Male	5Male	Frog	0	2.48	1.90	14.42	na	na
Male	5Male	Gecko	0	2.48	0.13	14.42	na	na
Male	5Male	Spider	1	2.74	0.59	14.42	78	1
Male	6Male	Spider	1	1.89	0.46	13.96	80	1
Male	6Male	Cockroach	1	2.22	0.90	13.96	53	5
Male	6Male	Gecko	1	1.87	0.22	13.96	78	2
Male	6Male	Frog	0	1.85	1.90	13.96	na	na
Male	7Male	Frog	0	2.18	1.02	13.44	na	na
Male	7Male	Gecko	1	2.55	0.41	13.44	99	3

Male	7Male	Spider	1	2.68	0.36	13.44	55	1
Male	7Male	Cockroach	0	2.60	0.65	13.44	na	na
Male	8Male	Frog	0	2.07	0.89	13.51	na	na
Male	8Male	Gecko	1	2.22	0.31	13.51	50	2
Male	8Male	Cockroach	1	2.44	1.00	13.51	58	1
Male	8Male	Spider	1	2.31	0.29	13.51	137	1
Male	9Male	Gecko	1	1.79	0.24	11.54	45	2
Male	9Male	Spider	1	2.02	0.49	11.54	94	4
Male	9Male	Frog	0	1.70	0.89	11.54	na	na
Male	9Male	Cockroach	0	1.70	0.63	11.54	na	na
Male	10Male	Gecko	1	1.53	0.23	8.58	71	1
Male	10Male	Cockroach	1	1.84	0.82	8.58	51	2
Male	10Male	Spider	1	1.86	0.22	8.58	88	1
Male	10Male	Frog	0	1.42	0.89	8.58	na	na
Male	11Male	Gecko	1	2.90	0.25	14.90	128	1
Male	11Male	Cockroach	0	2.72	0.87	14.90	na	na
Male	11Male	Spider	1	3.78	0.60	14.90	45	1
Male	11Male	Frog	0	2.76	1.90	14.90	na	na
Male	12Male	Spider	1	2.28	0.43	15.29	65	1
Male	12Male	Cockroach	0	1.99	0.87	15.29	na	na
Male	12Male	Gecko	0	1.99	0.13	15.29	na	na
Male	12Male	Frog	0	1.99	1.02	15.29	na	na
Male	13Male	Spider	1	2.11	0.67	14.48	51	1
Male	13Male	Gecko	0	2.15	0.17	14.48	na	na
Male	13Male	Cockroach	0	2.15	1.02	14.48	na	na
Male	13Male	Frog	0	2.15	1.23	14.48	na	na
Male	14Male	Gecko	1	2.05	0.29	13.36	49	1
Male	14Male	Spider	1	2.18	0.48	13.36	137	1
Male	14Male	Cockroach	1	1.96	0.90	13.36	104	1
Male	14Male	Frog	0	1.92	1.23	13.36	na	na
Male	15Male	Spider	1	1.70	0.39	11.02	39	5

Male	15Male	Cockroach	1	1.69	0.63	11.02	94	1
Male	15Male	Frog	0	1.55	0.89	11.02	na	na
Male	15Male	Gecko	1	1.68	0.23	11.02	22	1
Male	16Male	Spider	1	1.38	0.32	10.31	60	1
Male	16Male	Gecko	1	1.50	0.44	10.31	24	3
Male	16Male	Frog	0	1.27	0.89	10.31	na	na
Male	16Male	Cockroach	1	1.73	1.12	10.31	165	1
Male	17Male	Cockroach	1	2.21	0.99	12.59	66	1
Male	17Male	Spider	1	2.16	0.25	12.59	42	1
Male	17Male	Frog	0	1.89	0.89	12.59	na	na
Male	17Male	Gecko	1	2.13	0.49	12.59	46	1
Male	18Male	Cockroach	1	2.01	0.90	10.61	84	2
Male	18Male	Gecko	1	2.06	0.29	10.61	151	1
Male	18Male	Frog	0	2.15	1.03	10.61	na	na
Male	18Male	Spider	1	2.38	0.26	10.61	54	1
Male	19Male	Cockroach	1	1.37	0.70	9.22	145	2
Male	19Male	Spider	1	1.67	0.61	9.22	109	1
Male	19Male	Frog	0	1.60	1.03	9.22	na	na
Male	19Male	Gecko	1	1.82	0.34	9.22	229	2
Male	20Male	Cockroach	1	1.86	1.06	10.82	109	1
Male	20Male	Spider	1	1.93	0.18	10.82	141	1
Male	20Male	Gecko	1	1.82	0.22	10.82	111	1
Male	20Male	Frog	0	1.69	1.03	10.82	na	na
Female	1Female	Frog	0	2.38	1.12	12.01	na	na
Female	1Female	Cockroach	1	2.38	0.71	12.01	105	1
Female	1Female	Gecko	0	2.38	0.52	12.01	na	na
Female	1Female	Spider	0	2.38	0.49	12.01	na	na
Female	2Female	Spider	1	2.15	0.32	10.92	97	1
Female	2Female	Cockroach	0	2.15	0.42	10.92	na	na
Female	2Female	Frog	0	2.15	0.86	10.92	na	na
Female	2Female	Gecko	0	2.15	0.48	10.92	na	na

Female	3Female	Spider	1	3.06	0.38	16.25	64	1
Female	3Female	Cockroach	1	3.20	0.81	16.25	164	1
Female	3Female	Frog	0	3.55	1.33	16.25	na	na
Female	3Female	Gecko	1	3.55	0.26	16.25	86	3
Female	4Female	Gecko	1	2.29	0.27	13.70	77	1
Female	4Female	Frog	0	2.29	1.39	13.70	na	na
Female	4Female	Spider	0	2.29	0.27	13.70	na	na
Female	4Female	Cockroach	0	2.23	0.79	13.70	na	na
Female	5Female	Gecko	1	2.34	0.34	14.85	159	2
Female	5Female	Spider	0	2.34	0.28	14.85	na	na
Female	5Female	Cockroach	0	2.34	0.73	14.85	na	na
Female	5Female	Frog	0	2.34	1.39	14.85	na	na
Female	6Female	Spider	1	2.67	0.28	13.17	41	1
Female	6Female	Cockroach	0	2.67	1.15	13.17	na	na
Female	6Female	Frog	0	2.67	1.37	13.17	na	na
Female	6Female	Gecko	0	2.67	0.13	13.17	na	na
Female	7Female	Spider	1	2.67	0.43	15.91	74	1
Female	7Female	Frog	0	2.79	0.66	15.91	na	na
Female	7Female	Gecko	1	2.79	0.21	15.91	82	1
Female	7Female	Cockroach	1	2.76	0.81	15.91	41	1
Female	8Female	Gecko	1	3.22	0.29	16.48	131	1
Female	8Female	Cockroach	1	3.17	0.79	16.48	103	1
Female	8Female	Spider	1	3.45	0.39	16.48	33	1
Female	8Female	Frog	0	3.45	1.23	16.48	na	na
Female	9Female	Frog	0	3.45	1.47	14.34	na	na
Female	9Female	Gecko	1	3.45	0.26	14.34	52	1
Female	9Female	Spider	1	3.37	0.50	14.34	55	1
Female	9Female	Cockroach	1	3.43	1.08	14.34	14	1
Female	10Female	Spider	1	1.56	0.25	10.66	68	1
Female	10Female	Cockroach	1	1.68	0.79	10.66	49	1
Female	10Female	Gecko	1	2.16	0.15	10.66	112	1

Female	10Female	Frog	0	2.16	1.90	10.66	na	na
Female	11Female	Gecko	1	3.79	0.21	15.10	78	2
Female	11Female	Spider	1	3.57	0.34	15.10	86	1
Female	11Female	Cockroach	1	3.50	0.64	15.10	152	1
Female	11Female	Frog	0	3.50	1.90	15.10	na	na
Female	12Female	Spider	1	2.18	0.64	12.59	33	1
Female	12Female	Cockroach	1	2.35	1.17	12.59	40	1
Female	12Female	Gecko	1	2.66	0.67	12.59	31	1
Female	12Female	Frog	0	2.66	1.90	12.59	na	na
Female	13Female	Spider	1	2.32	0.71	13.74	47	1
Female	13Female	Cockroach	1	2.44	0.88	13.74	62	1
Female	13Female	Frog	0	2.84	0.89	13.74	na	na
Female	13Female	Gecko	1	2.84	0.31	13.74	140	4
Female	14Female	Gecko	0	2.67	0.17	15.98	na	na
Female	14Female	Spider	1	2.67	0.63	15.98	31	4
Female	14Female	Cockroach	1	2.85	0.96	15.98	171	1
Female	14Female	Frog	0	2.81	1.39	15.98	na	na
Female	15Female	Gecko	1	1.18	0.20	11.51	76	2
Female	15Female	Cockroach	1	1.16	0.69	11.51	311	1
Female	15Female	Spider	1	1.39	0.24	11.51	59	3
Female	15Female	Frog	0	1.39	1.90	11.51	na	na
Female	16Female	Spider	1	2.53	0.28	16.39	58	1
Female	16Female	Frog	0	2.64	1.39	16.39	na	na
Female	16Female	Gecko	0	2.64	0.21	16.39	na	na
Female	16Female	Cockroach	0	2.60	0.79	16.39	na	na
Female	17Female	Frog	0	1.75	1.03	13.01	na	na
Female	17Female	Cockroach	0	1.75	1.10	13.01	na	Na
Female	17Female	Gecko	0	1.75	0.69	13.01	na	Na
Female	17Female	Spider	1	1.75	0.53	13.01	24	1
Female	18Female	Frog	0	2.06	1.23	14.35	na	Na
Female	18Female	Spider	1	2.06	0.27	14.35	52	1

Female	18Female	Cockroach	1	2.04	0.59	14.35	175	1
Female	18Female	Gecko	1	2.09	0.32	14.35	46	1
Female	19Female	Spider	1	1.46	0.56	11.59	85	1
Female	19Female	Cockroach	1	1.55	1.08	11.59	48	1
Female	19Female	Frog	0	1.55	1.29	11.59	na	Na
Female	19Female	Gecko	0	1.55	0.43	11.59	na	Na
Female	20Female	Cockroach	1	1.04	0.74	9.45	68	2
Female	20Female	Gecko	1	1.31	0.17	9.45	49	1
Female	20Female	Frog	0	1.04	1.01	9.45	na	Na
Female	20Female	Spider	0	1.04	0.54	9.45	na	Na

Table S2. Mean volume (µL) of venom produced in both cheliceral and prosoma length (mm) by males and females of *P. boliviensis*.

Sex	Mean Volume	Prosoma Length
Male	2.81	12.73
Male	6.83	13.84
Male	0.29	12.64
Male	4.49	14.52
Male	1.39	12.78
Male	2.02	15.28
Male	2.82	11.30
Male	2.71	13.84
Male	3.35	9.54
Male	3.78	11.88
Male	3.88	10.87
Male	4.10	11.22
Male	3.59	12.92
Male	3.65	10.98
Male	4.00	12.17
Female	24.54	12.48
Female	13.49	15.50
Female	1.99	13.79
Female	12.71	15.60
Female	2.72	11.47
Female	18.56	15.95
Female	4.83	13.40
Female	14.52	12.12
Female	5.75	14.10
Female	5.08	15.91
Female	11.35	11.81
Female	1.33	12.76
Female	7.53	12.77
Female	12.80	13.45
Female	5.41	11.79
Female	6.71	15.59
Female	2.60	11.78
Female	2.93	11.46

Table S3. List of doses (mg of venom/kg of prey) used the in the bioassays for females (F) and males (M) of *P. boliviensis* and two prey types, gecko (*Hemidactylus frenatus*) and spider (*Spinoctenus* sp.). (Dead=number of dead individuals after venom application).

Dose (mg/kg)	Prey	Sex	N° of Individuals	Dead
100	*H. frenatus*	F	6	6
30	*H. frenatus*	F	10	10
15	*H. frenatus*	F	10	10
10	*H. frenatus*	F	6	5
8	*H. frenatus*	F	10	8
5	*H. frenatus*	F	10	10
4.5	*H. frenatus*	F	10	8
3.5	*H. frenatus*	F	10	9
3	*H. frenatus*	F	10	4
2.5	*H. frenatus*	F	10	7
2.3	*H. frenatus*	F	15	8
1	*H. frenatus*	F	6	1
0.3	*H. frenatus*	F	10	0
0.03	*H. frenatus*	F	10	0
0	*H. frenatus*	F	46	0
2200	*Spinoctenus* sp.	F	10	7
2000	*Spinoctenus* sp.	F	10	6
1800	*Spinoctenus* sp.	F	10	3
1200	*Spinoctenus* sp.	F	10	4
1000	*Spinoctenus* sp.	F	10	2
500	*Spinoctenus* sp.	F	10	2
100	*Spinoctenus* sp.	F	10	1
10	*Spinoctenus* sp.	F	10	0
1	*Spinoctenus* sp.	F	10	2
0	*Spinoctenus* sp.	F	30	0
4.5	*H. frenatus*	M	10	10
3.5	*H. frenatus*	M	10	9
3	*H. frenatus*	M	10	8
2.5	*H. frenatus*	M	10	8
2	*H. frenatus*	M	10	5
1.5	*H. frenatus*	M	10	2
0	*H. frenatus*	M	20	0
2000	*Spinoctenus* sp.	M	10	7
1800	*Spinoctenus* sp.	M	10	8
1500	*Spinoctenus* sp.	M	10	5
1200	*Spinoctenus* sp.	M	10	6
500	*Spinoctenus* sp.	M	10	3
0	*Spinoctenus* sp.	M	20	0
